# Reflecting on the methodological challenge of recruiting older care home residents to podiatry research

**DOI:** 10.1186/1757-1146-8-S1-A10

**Published:** 2015-04-20

**Authors:** Gavin Wylie, Zoë Young, Roberta Littleford, Frank Sullivan, Brian Williams, Hylton Menz, Simon Ogston, Jacqui Morris

**Affiliations:** 1 Department of Podiatry, Allied Health Professions Directorate, NHS Tayside, Dundee, UK; 2 Social Dimensions of Health Institute, Dundee University, Dundee, UK; 3 Tayside Medical Science Centre, Ninewells Hospital and Medical School, Dundee, UK; 4 Department of Family & Community Medicine, University of Toronto, North York General Hospital, Canada; 5 Nursing Midwifery and Allied Health Professions Research Unit, Stirling University, UK; 6 Lower Extremity and Gait Studies Program, La Trobe University, Australia; 7 Centre for Biomedical Science and Public Health, University of Dundee, Dundee, UK; Social Dimensions of Health Institute, Dundee University, UK

## Introduction

Successful randomisd controlled trials (RCTs) require successful participant recruitment; poor recruitment leads to poor, under-powered studies, and may waste grant funds. Recruitment of older care home residents to RCTs is challenging. This is problematic for podiatry, because older people within care home settings are high users of podiatry services; therefore it is essential that strategies are employed to maximise recruitment to RCTs.

We describe the experience of recruiting to a feasibility study of a podiatry intervention to reduce falls in care home residents in the East of Scotland. This was the first phase of a two phase project consisting of the feasibility study to acquire data (recruitment strategy, selection of suitable outcome measures) to inform the conduct of the second phase, an exploratory RCT. Recruitment difficulties became apparent early in the study. Difficulties arose when it came to assessing whether or not potential participants fulfilled certain inclusion criteria:

(1) Presence of foot pain (defined as foot pain lasting for at least a day in the last month and a positive response of “some days” or “most/every days” to at least one item on the Manchester Foot Pain and Disability Index (MFPDI))

(2) Ability to provide informed consent.

The reasons for these difficulties are that (1) we discovered that in the area in which our study was conducted, the majority of care home residents receive basic NHS podiatry care to treat any superficial lesions (i.e. pathological nails and skin callus) thus the prevalence of foot pain resulting from these lesions was lower than we had originally anticipated, and (2) the care homes that we engaged for this phase of the study had residents who were far more dependent and with much higher levels cognitive impairment than we anticipated, making obtaining informed consent difficult. Based on the existing inclusion criteria, it was deemed unlikely that we would meet our recruitment target for the subsequent exploratory randomised controlled trial (n=40).

## Methods

Following discussion with co-applicants we proposed to make two changes in order to improve recruitment, whilst maintaining the scientific integrity of the protocol:

(1) We engaged with care homes that cater for less dependent residents in order to improve the likelihood of obtaining informed consent.

(2) Since evidence shows that there are several foot and ankle characteristics (toe muscle weakness, hallux valgus, decreased ankle flexibility and strength) that are associated with falls but which do not necessarily cause pain, we widened the inclusion criteria by removing foot pain as a criterion. The recruitment difficulties required a 3 month prolongation of the study duration.

## Results

As a result of tailoring the recruitment strategy early in the feasibility study, we recruited rapidly to the exploratory RCT. We have exceeded our target (n=48).

## Conclusions

Care home residents represent a convenient population for data collection, but frailty and multiple co-morbidities may make successful recruitment to intervention studies challenging. Whilst the adaptations used in this study may have implications for external validity, this work underlines the importance of testing recruitment strategies at an early stage.

